# Shaping the Future of Probiotics: Novel Methodologies, Applications, and Mechanisms of Action

**DOI:** 10.3390/microorganisms12010073

**Published:** 2023-12-29

**Authors:** Alex Galanis

**Affiliations:** Department of Molecular Biology and Genetics, Faculty of Health Sciences, Democritus University of Thrace, 68100 Alexandroupolis, Greece; agalanis@mbg.duth.gr

Probiotics are defined as live microorganisms that, when consumed in appropriate amounts, can promote host homeostasis, and induce health-promoting effects [1]. Bifidobacteria and members of the phylogenetically diverse, emended *Lactobacillus* genus are commonly classified as probiotics due to their safety profile and potential health benefits. Probiotics have been historically linked with gastrointestinal health; however, accumulating literature suggests that they can also exert their beneficial effects on distant tissues and organs [2]. Mechanistic studies show that at the basis of their action lies their ability to induce immunomodulatory, immunostimulatory, antioxidant, antimicrobial and antibiofilm action [3]. In this Special Issue of *Microorganisms*, four research articles deal with different aspects of probiotic activity, including their antagonistic activity against food-borne pathogens [4], and the application of omics technologies, such as transcriptomics and metabolomics, to study the metabolic and physiological properties of probiotic strains in different food matrices [5]. Moreover, three review articles in this Special Issue summarize recent experimental findings in probiotic research, focusing on the main mechanisms of action, and critically discuss the potential beneficial effects of probiotics in human [6,7], as well as in animal, plant, soil, and environmental health, in the context of the ‘’One Health’’ approach [8].

In vitro and in vivo assays as well as in silico algorithms are commonly employed to study strain-specific properties of novel, potential probiotic isolates. The next big challenge will be to translate these findings into the clinic to achieve targeted preventative or therapeutic probiotic interventions with increased efficacy. Moreover, the investigation and elucidation of the spatiotemporal probiotic–host interactions through multi-omic platforms are required. The critical role of the gut microbiota in these interactions should also be defined. Future studies should also focus on identifying bioactive compounds in probiotics (postbiotics) and characterizing their mechanisms of action to further elucidate their biological effects.
